# Evaluation of pretreatment with Cetrotide in an antagonist protocol for patients with PCOS undergoing IVF/ICSI cycles: a randomized clinical trial

**DOI:** 10.5935/1518-0557.20180039

**Published:** 2018

**Authors:** Maryam Eftekhar, Ramesh Baradaran Bagheri, Nosrat Neghab, Robabe Hosseinisadat

**Affiliations:** 1 Reasearch and Clinical Center for Infertility, Yazd Reproductive Sciences Institute, Shahid Sadoughi University of Medical Sciences, Yazd, Iran.; 2 Recurrent Abortion Research Center, Yazd Reproductive Sciences Institute, Shahid Sadoughi University of Medical Sciences, Yazd, Iran.; 3 Department of Obstetrics and Gynecology, School of Medicine, Kerman University of Medical Sciences, Kerman, Iran.

**Keywords:** assisted reproductive technology, gonadotropins, polycystic ovarian syndrome

## Abstract

**Objective:**

This study aimed to evaluate the effect of three days of GnRH antagonist pretreatment on the pregnancy outcomes of women with polycystic ovarian syndrome (PCOS) on GnRH antagonist protocols for IVF/ICSI.

**Methods:**

Fifty women with PCOS in the control group received conventional antagonist protocols, starting on day 2 of the cycle. In the pretreatment group (n=38), a GnRH antagonist was administered from day 2 of the menstrual cycle for three days.

**Results:**

Controlled ovarian stimulation (COS) duration and gonadotropin dosages were similar in both groups. The number of metaphase II (MII) oocytes, 2PN oocytes, embryos, along with implantation and clinical pregnancy rates, were higher in the pretreatment group when compared with controls, although the increment was not significant (*P* value ≥0.05). The chemical pregnancy rate was significantly higher in the pretreatment group. The rate of OHSS was significantly lower in the pretreatment than in the control group.

**Conclusion:**

Women with PCOS offered early follicular phase GnRH antagonist pretreatment for three consecutive days had significantly fewer cases of OHSS and higher chemical pregnancy rates. There were trends toward greater numbers of MII oocytes, 2PN oocytes, and embryos, and higher clinical pregnancy rates in the pretreatment group.

## INTRODUCTION

Polycystic ovary syndrome (PCOS) is a very common endocrine disorder. It affects 5-7% women of reproductive age and is the leading cause of anovulatory infertility in this age range (Singh *et al.*, 2014; Kalem *et al*., 2017). Irregular menstruation, hirsutism, acne, and infertility are common clinical features. Forty percent of infertile women have anovulation/oligoovulation and PCOS accounts for 80% of these cases (Singh *et al.*, 2014). Assisted reproductive technology (ART) protocols are indicated when infertile women with PCOS are unable to become pregnant through standard ovulation induction methods. Ovarian stimulation includes two methods to prevent premature LH surges, GnRH agonist and GnRH antagonist protocols (Toftager *et al*., 2016). However, issues such as increased risk of ovarian hyperstimulation syndrome (OHSS), increased immature oocyte rates, lower fertilization rates, lower embryo quality, and lower implantation rates are observed in the IVF cycles of women with PCOS when the two protocols are compared (Kalem *et al*., 2017).

GnRH antagonists have been extensively used in ART clinics during the past years and a variety of GnRH antagonist protocols have been suggested. In spite of GnRH agonist protocol cycles, GnRH antagonists cause immediate suppression of gonadotropin secretion, which results in shorter treatments and less patient distress (European and Middle East Orgalutran Study Group, 2001; Al-Inany *et al*., 2006; Devroey *et al*., 2009). Moreover, the use of GnRH antagonists has yielded significantly lower chances of hospitalization due to OHSS (Kolibianakis *et al.*, 2006). Despite the benefits associated with GnRH antagonists, GnRH agonist protocols remain as the treatment of choice in controlled ovarian hyperstimulation (COH) in the majority of ART clinics. There are some reasons for this practice. First, some investigators have reported uncoordinated antral follicle growth during ovarian stimulation with GnRH antagonists, leading to an asynchrony of the follicular cohort (Fanchin *et al.*, 2003), which in turn may raise concerns over the outcome of the treatment. The flexibility of GnRH agonist protocols also permits more controlled oocyte retrievals, significantly decreasing and even preventing the need to perform retrievals, whereas the initiation of ovarian stimulation in GnRH antagonist protocol relies on the random incidence of spontaneous menses (Guivarc'h-Levêque *et al.*, 2010; Levy *et al.*, 2009; Tremellen & Lane, 2010). Therefore, pretreatment with oral contraceptive pills (OCP) is frequently used in GnRH antagonist protocols to schedule the start of gonadotropin stimulation, although it considerably increases the consumption of gonadotropins and the duration of ovarian stimulation (Griesinger *et al.*, 2008). A recent meta-analysis detected a considerable decrease in the ongoing pregnancy rate of patients prescribed pretreatment with OCP (Griesinger *et al.*, 2010). Comparisons between a pituitary down-regulation protocol and a GnRH antagonist protocol at the beginning of ovarian stimulation found that women with PCOS in particular had higher serum gonadotropin and E2 levels. Consequently, in these women the unsuppressed level of FSH at the start of a GnRH antagonist cycle in contrast with a long GnRH agonist protocol allows the initial growth of a few leading follicles before the addition of exogenous rFSH (Blockeel *et al.*, 2011a). We hypothesized that short pituitary suppression in the early follicular phase might mimic the pre-stimulation hormonal environment of long GnRH agonist protocols, challenging the idea that high endogenous FSH levels cause developmental asynchrony of early antral follicles while maintaining the benefits of short antagonist protocols.

Furthermore, stable and early suppression of LH levels during the entire period of stimulation may be advantageous for implantation and pregnancy outcomes (Blockeel *et al.*, 2011a). Occasional elevated baseline progesterone levels at the beginning of ART cycles and their association with reduced pregnancy outcomes is another problem in GnRH antagonist protocols (Kolibianakis *et al.*, 2004a; Blockeel *et al.*, 2011b). Based on these data, administration of GnRH antagonists for three consecutive days before the start of COS may normalize raised progesterone levels.

In agreement with the above findings, we posited that pretreatment with GnRH antagonists might allow follicular cohort synchronization and scheduling of ART treatment in women with PCOS. The purpose of this prospective randomized trial was to evaluate the effects of a 3-day course of GnRH antagonist pretreatment before the initiation of ovarian stimulation with gonadotropins on pregnancy outcomes.

## MATERIALS AND METHODS

### Study Design

This randomized clinical trial enrolled 88 women with PCOS based on the Rotterdam criteria, participating in an ART program at the Yazd Research and Clinical Center for Infertility, Shahid Sadoughi University of Medical Sciences, from March 2015 to March 2016. The Ethics Committee of the university approved the study. Informed written consent was obtained from all participating couples.

Patients with at least two of the following findings were included in the study: oligoovulation or anovulation; clinical or biochemical hyperandrogenism; and polycystic ovaries on ultrasound examination. Women aged 40 years or older, presence of severe male factor or systemic disease, use of hormone medication other than OCP, individuals on systemic drug therapy or with recurring IVF failure, recurrent pregnancy loss or uterine anomalies were excluded.

**Figure 1:**
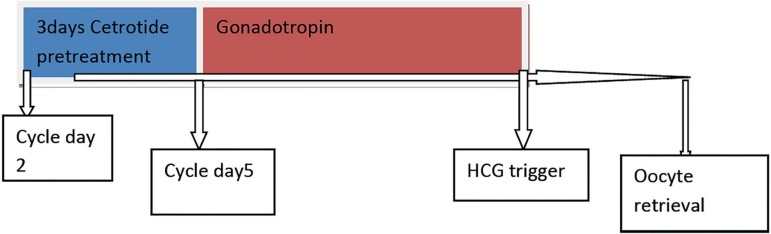
Schematic view of cetrotide pretreatment protocol.

Two GnRH antagonist protocols for ovarian stimulation were compared. The patients were randomly (Random Digit Software) allocated to two groups. The 50 individuals assigned to the control group were prescribed a standard GnRH antagonist protocol. Controls were administered Gonal-f 150 IU (SA Merck Serono, Geneva, Switzerland) on cycle day 2 subcutaneously, and later 0.25 mg of cetrorelix (Cetrotide; Asta Medica, Frankfurt, Germany) daily when the leading follicle reached 14 mm in diameter until the HCG injection. The 38 women allocated in the pretreatment group were offered a modified protocol with antagonist administration for three days (starting on day 2 of the cycle) before the start of recombinant FSH (rFSH) therapy (Figure 1). Final oocyte maturation was triggered with HCG 10,000 IU (Pregnyl; Schering Plough) when the three larger follicles reached a mean diameter of 17 mm. Serum estrogen (E2) and endometrial thickness (ET) were measured on triggering day. Ultrasound-guided transvaginal oocyte retrieval was performed 36 hours later. Follicles measuring ≥14 mm were aspirated and the physicians performing follicular aspiration were blinded to the stimulation protocol. The IVF and ICSI procedures were performed, and the embryos were transferred on the third day after retrieval with a catheter (Labotect, Gotting, Germany). Embryo quality was assessed based on the morphology criteria described by Dokras *et al.*; cleavage-stage embryos were given grades A, B, C, or D. Embryos graded as D were not transferred. Grade A included embryos with no fragmentation and equal size homogenous blastomeres; grade B included embryos with fragmentation <20% and equal size homogenous blastomeres; grade C included embryos with fragmentation ranging from 20% to 50% and unequal size blastomeres; grade D included embryos with fragmentation >50% and unequal size blastomeres (Dokras *et al.*, 1993).

The number of transferred embryos depended on embryo quality, patient age, and risk of OHSS. Women at moderate or severe risk of OHSS had their embryos frozen as described in Table 1.

**Table 1:** Classification of OHSS.

Grade	Symptom
Mild OHSS	Abdominal bloating
	Mild abdominal pain
	Ovarian size usually <8 cm
Moderate OHSS	Moderate abdominal pain
	Nausea ± vomiting
	Ultrasound evidence of ascites
	Ovarian size usually 8 to 12 cm
Severe OHSS	Clinical ascites (occasionally pleural effusion)
	Oliguria
	Hematocrit (>45%)
	Hypoproteinemia
	Ovarian size usually >12 cm

All patients were given 400 mg progesterone suppositories (Cox Pharmaceuticals, Barnstaple, UK) twice a day for luteal support, initiated on the day of oocyte retrieval. Serum B-hCG was checked 14 days after embryo transfer. If the patient became pregnant, then progesterone was continued until the 10^th^ week of pregnancy. The implantation rate was calculated as the ratio between the number of gestational sacs and transferred embryos; chemical pregnancy was defined by serum B-hCG levels ≥50 IU/L 14 days after embryo transfer; clinical pregnancy was established as the presence of a gestational sac with fetal heartbeat identified by ultrasound examination five weeks after embryo transfer; miscarriages were defined as clinically recognized pregnancy losses before 20 weeks of gestation; and ongoing pregnancies were defined as pregnancies continued after 20 weeks of gestation.

**Figure 2:**
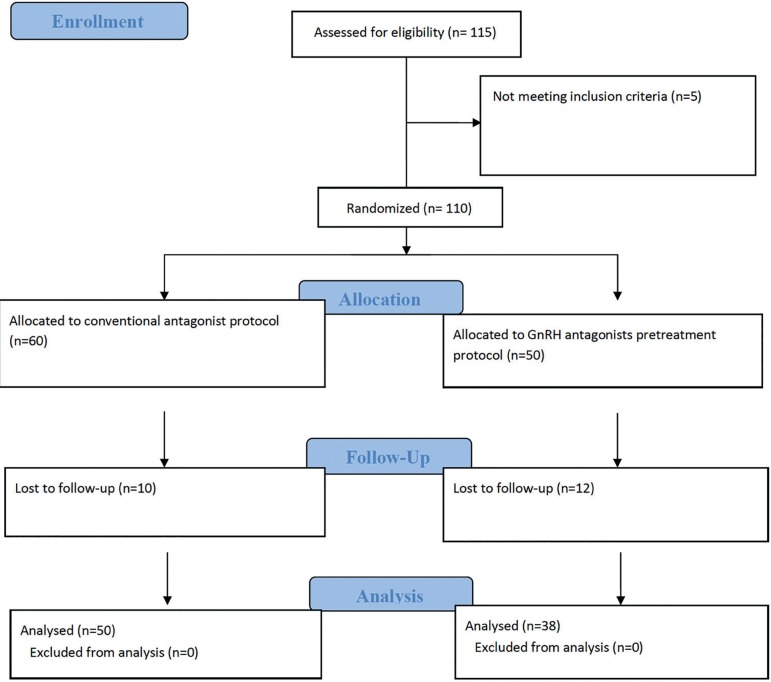
Consort flowchart.

The two groups had different sizes on account of patients lost to follow-up (Figure 2).

### Outcome Measures

The primary endpoints in this study were the number of cumulus oocyte complexes (COCs), metaphase II oocytes (MII), and 2-pronuclei (2PN) oocytes in each group. Secondary endpoints included fertilization, implantation, and pregnancy rates in each treatment group. Tertiary endpoints were risk of OHSS and miscarriage rates in each group. Demographic and clinical characteristics such as age, baseline serum hormone levels, AMH, duration of stimulation, and total cumulative dose of rFSH were also collected.

### Statistical Analysis

SPSS software (Statistical Package for the Social Sciences version 16.0, SPSS Inc., Chicago, IL, USA) was used for all statistical calculations. Student's t-test was used to compare quantitative variables. Statistical significance was attributed to differences with a *p* value<0.05.

## RESULTS

Eighty-eight patients were randomly assigned to either control (n=50) or pretreatment groups (n=38). A flowchart diagram with the phases of the trial is shown in Figure 2. Baseline characteristics of the study groups are presented in Supplemental Table 2. The groups did not significantly differ with regard to demographic and cycle parameters. Table 2 summarizes the outcome parameters of both treatment protocols. Embryos from 18 women in the control group and 15 in the pretreatment group were frozen due to risk of OHSS. The implantation rates in the pretreatment and control groups were 20.5%±27.3% and 11.8%±20.6%, respectively. The difference was not statistically significant. Chemical and clinical pregnancy rates in the pretreatment group were higher (*p*˂0.05) (Table 3). Ongoing pregnancy rates were higher in the pretreatment group, although not statistically different from the rates seen in the control group (Table 3).

**Table 2:** Baseline and cycle characteristics of patients in both groups.

	Pretreatment group (n=38)	Control group (n=50)	*p*-value
Age (years)	28.07±3.85	29.04±4.76	.313
Duration of infertility (years)	6.57±3.77	5.99±3.74	.468
AMH	6.64±1.29	6.46±1.01	.367
End. Thickness at triggering day (mm)	10.27±1.92	10.15±1.98	.770
Estradiol level at triggering day (ng/ml)	3323±2670	2967±2149	.491
Gonadotropin Dose (IU)	1598±1932	1515±1475	.771
Cycle duration (days)	12.36±1.54	13.66±2.23	.003
COC number	17.68±9.29	16.46±9.76	.554
Number of M2 oocytes	14.65±8.30	14.10±8.79	.764
Number of 2PN oocytes	8.84±6.67	7.40±6.41	.308
Total number of embryos	7.94±6.16	6.94±6.06	.446
Number of embryos transferred	2.80±0.7	2.69±0.6	.676
Number of embryos frozen	3.56±2.10	4.13±2.01	.454

Data are presented as mean value ± SD or number (%).

**Table 3:** Pregnancy outcomes of patients in both groups.

	Pretreatment group (n=38)	Control group (n=50)	*p*-value
Fertilization rate	51.7%	50.6%	0.49
Implantation rate	20.5±27.3%	11.8±20.6%	.091
Chemical pregnancy rate (n, %)*	9 (41%)	3 (13%)	.035
Clinical pregnancy rate (n, %)*	7 (32%)	2 (9%)	.050
Ongoing pregnancy rate (n, %)	6 (28%)	2 (9%)	.103
Moderate, severe risk of OHSS (n, %)	15 (39%)	18 (36%)	.739
Miscarriage rate (n, %)	3 (37%)	1 (33%)	.898

* Student’s *t* test.

## DISCUSSION

The study showed that pretreatment with GnRH antagonists for three consecutive days before the start of ovarian stimulation tends to yield a greater number of COCs when compared with conventional GnRH antagonist protocols. Although not significant, a greater number of 2PN oocytes and higher pregnancy rates were also observed in the pretreatment group. Using a similar protocol with GnRH antagonist pretreatment, Younis *et al.* (2010) reported improved oocyte maturation and fertilization rates. Although our study included only 38 patients in pretreatment, potential improvements in clinical outcomes might be inferred.

Huirne *et al.* (2007) suggested that early suppression of endogenous FSH results in improved follicular development. In a GnRH antagonist protocol, higher serum gonadotropin and estradiol levels are found at the beginning of ovarian stimulation, when compared with long GnRH agonist protocols (Albano *et al.*, 2000; Borm & Mannaerts, 2000). As a result, the unsuppressed FSH level at the beginning of a GnRH antagonist cycle allows the initial growth of a few leading follicles before the initiation of exogenous rFSH, in contrast with a long GnRH agonist protocol. Pretreatment with estrogen or OCP in GnRH antagonist regimens offers a simple alternative to achieve endogenous gonadotropin suppression during the early follicular phase (de Ziegler, 1995; van Heusden & Fauser, 1999). In order to challenge the idea that elevated endogenous FSH levels cause developmental asynchrony of early antral follicles, Fanchin *et al.* (2003) posited that "luteal phase E2 pretreatment and premenstrual administration of GnRH antagonist can reduce the size and improve the homogeneity of early antral follicles". We propose that short pituitary suppression in the early follicular phase might mimic the hormonal environment of long GnRH agonist protocols, while protecting the benefits of short antagonist protocols. Additionally, stable and early suppression of LH levels during the period of stimulation may be advantageous for implantation and pregnancy outcomes (Kolibianakis *et al.*, 2003). Kolibianakis *et al.* (2003; 2004b) demonstrated that "high exposure of the genital tract to LH and E2 in the early follicular phase is associated with a lower chance of pregnancy". As a corollary to this study, the same researchers concluded that giving a GnRH antagonist from day 1 of stimulation is an effective protocol that leads to effective results (implantation rate of 26.5%; ongoing pregnancy rate of 39.7% per started cycle and of 42.4% per ET).

On the day of hCG administration, significantly higher E2 levels were found in the pretreatment group, which may be explained by the increased recruitment of oocytes in this group. Although supraphysiological serum E2 levels might lead to adverse effects on oocyte/embryo quality and to impaired endometrial receptivity, higher E2 levels on the day of hCG administration have no impact on pregnancy rates in GnRH antagonist protocols (Kyrou *et al.*, 2009). Similarly, progesterone levels were significantly elevated on the last day of stimulation in the pretreatment group, but not to the extent that it might have a detrimental effect on the implantation potential of a good-quality cleavage-stage embryo (Bosch *et al.*, 2010).

The clinical potential of this modified short patient-friendly protocol is associated with a small financial cost per cycle. A possible problem of this pretreatment protocol is the addition of three SC injections. Although this trial suggests promising results in that a trend toward higher pregnancy rates was detected in the GnRH antagonist pretreatment group, statistical power analysis might be helpful to define the sample size needed to add weight to this observation. This approach might also be accepted for the added convenience of enabling scheduled GnRH antagonist cycles and for optimizing the organization of ART centers through the administration of GnRH antagonist cycles for a varying number of days (2, 3 or 4 days), based on the planned start of ovarian stimulation. This protocol might be used with oocyte donors to help synchronization with recipients. The additional advantage of triggering final oocyte maturation with a GnRH agonist is that it allows patients to avoid OHSS.

## CONCLUSION

In conclusion, pretreatment with GnRH antagonists for women with PCOS for three consecutive days before the beginning of ovarian stimulation was associated with improved pregnancy results. Further investigation is needed to find whether GnRH antagonist pretreatment leads to better coordination of multifollicular development in high responders.
